# The PfK13 G533S mutation confers artemisinin partial resistance in multiple genetic backgrounds of *Plasmodium falciparum*

**DOI:** 10.1128/aac.00162-25

**Published:** 2025-05-27

**Authors:** Faiza Amber Siddiqui, Aongruk Chim-Ong, Chenqi Wang, Jun Miao, Liwang Cui

**Affiliations:** 1 Department of Internal Medicine, Morsani College of Medicine, University of South Florida 33697, Tampa, Florida, USA; 2 Center for Global Health and Infectious Diseases Research and USF Genomics Program, College of Public Health, University of South Florida 27117, Tampa, Florida, USA; The Children's Hospital of Philadelphia, Philadelphia, Pennsylvania, USA

**Keywords:** *Plasmodium falciparum*, PfK13, artemisinin resistance, gene editing, ring-stage survival

## Abstract

Mutations in the *Plasmodium falciparum* Kelch 13 (PfK13) protein are the key determinant of artemisinin partial resistance. While more than 200 PfK13 mutations have been identified in global parasite populations, only 13 have been validated to confer *in vivo* or *in vitro* artemisinin partial resistance. In the western Greater Mekong Subregion, the prevalence of the PfK13 G533S mutation has significantly increased in recent years. Field isolates carrying the PfK13 G533S mutation showed slower parasite clearance and higher day-3 positivity rates after artemisinin treatment, while culture-adapted isolates displayed significantly elevated ring-stage survival rates. Here, the PfK13 G533S mutation was introduced using CRISPR/Cas9 into four parasite strains: Dd2, 3D7, GB4, and F09N25 (a recent culture-adapted field isolate from the China-Myanmar border area). Across all four genetic backgrounds, the PfK13 G533S mutation conferred ring-stage survival rates of 12%–23% with a minimal fitness cost, explaining its rising prevalence in the region. In contrast, the PfK13 G533A mutation, sporadically detected in world *P. falciparum* populations, did not increase ring-stage survival rates when engineered into the 3D7 and Dd2 strains. These findings validate the *PfK13* G533S mutation as a critical marker for artemisinin resistance surveillance and underscore the importance of monitoring its spread.

## INTRODUCTION

The progress toward malaria elimination is severely hampered by the emergence and expansion of *Plasmodium falciparum* parasites resistant to the first-line treatment of artemisinin (ART)-based combination therapies (ACTs). ART partial resistance is clinically manifested as delayed parasite clearance with a half-life of >5.5 h or persistence of parasites after three days of the standard ACT treatment (1, 2), which is captured by the *ex vivo* and *in vitro* ring-stage survival assay (RSA) (3). With the RSA, parasites with ART partial resistance show >1% survival rates when early ring-stage parasites are exposed to 6 h of 700 nM dihydroartemisinin (DHA), the active metabolite of ARTs (3, 4).

The initial reports of delayed parasite clearance came from Cambodia almost two decades ago (5, 6), followed by the detection of ART partial resistance in all countries of the Greater Mekong Subregion (GMS) (2). The primary determinant of ART partial resistance is the mutations in the propeller domain of the *P. falciparum* Kelch 13 (PfK13) protein (4). Over 200 mutations have since been reported in PfK13 (7), but only about 20 are validated or candidates for association with ART partial resistance (8). Recently, PfK13 mutations have emerged *de novo* in East Africa, where they have become established (9–12). Several mutations, including R561H, C469Y, R622I, and A675V, have been validated through the gene editing analysis (13–15). This knowledge is important for guiding resistance surveillance to track the appearance and dispersion of ART partial resistance.

Our efforts to track the dynamics of PfK13 mutations in Myanmar led to the identification of a new mutation, G533S, which was not detected in the China-Myanmar border region before 2013 but rose to a high prevalence of 44% in culture-adapted clinical samples collected in 2014–2016 (16). This mutation was also detected in 10.8% of clinical samples from a DHA-piperaquine (PPQ) efficacy study conducted in the same border area from 2014 to 2018 (17). The G533S mutation was previously reported to be present sporadically in Cambodia (4, 14). Similarly, it was also detected in eastern Myanmar at a low prevalence of ~1% in clinical samples during six years (2013–2019) of mass drug administration with DHA-PPQ (18). However, in western Thailand bordering Myanmar, this mutation was detected at an increasing frequency from 20% in 2014 to 100% in 2019 (19). This suggests that the malaria situations at the China-Myanmar border and western Thailand may favor the selection of the G533S mutation. Outside of Southeast Asia, the G533S mutation was detected at a low prevalence in Zambia (7). Besides, different alleles for this codon were also reported at very low frequencies, such as G533A in India (20), Uganda (21), and Myanmar (18), G533C in Uganda (22), G533V in Senegal (23), and G533D in Myanmar (18).

Clinical efficacy studies of DHA-PPQ in the China-Myanmar border area linked the G533S mutation to a parasite clearance half-life of >5 h and day-3 positivity (17). Using culture-adapted parasite strains from this region, we also found that parasites carrying the G533S mutation had significantly higher RSA values than those carrying the wild-type (WT) allele (16). To validate whether this PfK13 mutation mediates ART partial resistance, we introduced G533S in four different genetic backgrounds using CRISPR/Cas9. We found that G533S led to higher RSA values in all the parasites tested without a significant effect on parasite fitness.

## RESULTS

### PfK13 G533S mutation is associated with ART partial resistance

**Fig 1:**
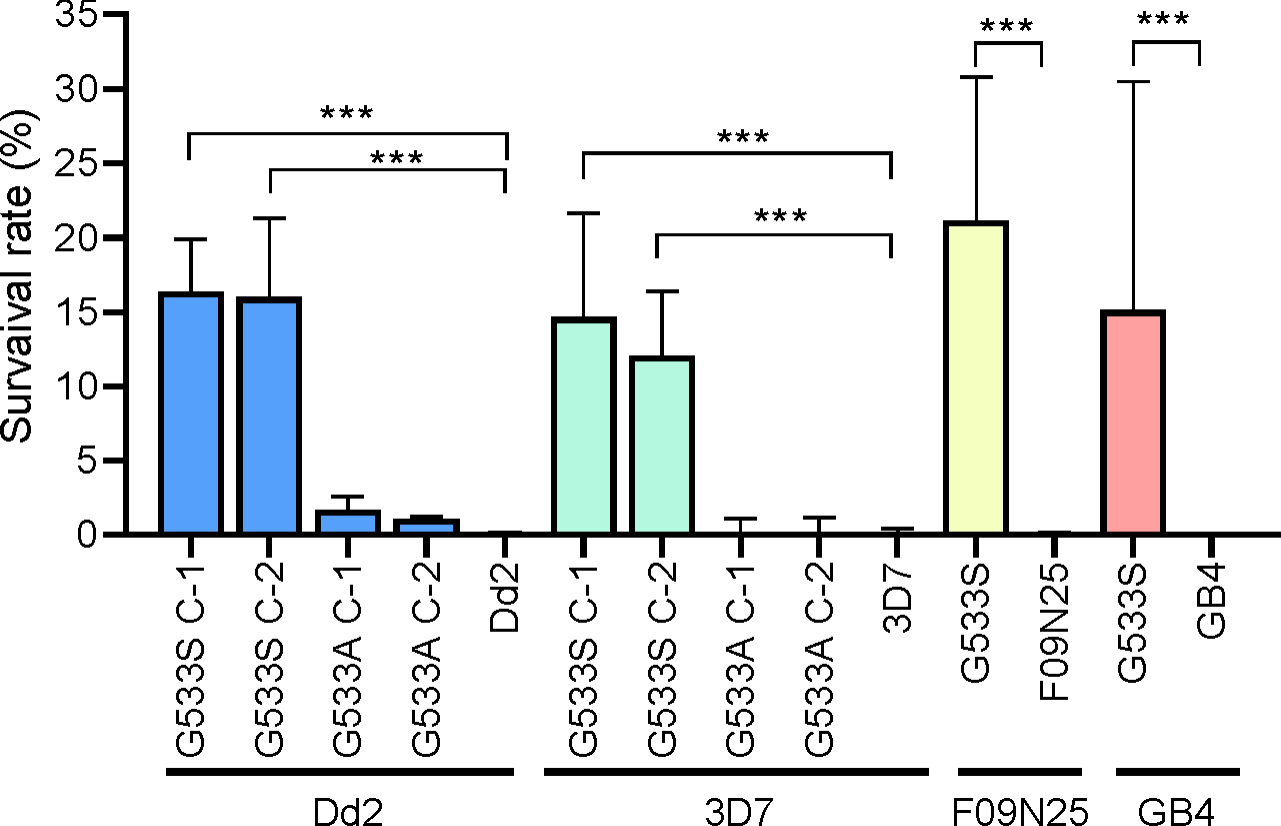
Ring stage survival rate (%) of parasite lines edited to carry the PfK13 G533S/A mutation. The G533S mutation was introduced into 3D7, Dd2, GB4, and F09N25 using CRISPR/Cas9. The G533A variation was also introduced in 3D7 and Dd2. 0–3 h rings were exposed to 700 nM DHA, and survival was measured at 72 h. Each experiment was performed in at least three biological replicates, and the mean and standard deviation for each survival rate value are shown. DMSO-treated parasites were used as the vehicle control. The survival rates of mutant parasite lines were compared with their respective wild-type parasites, and *** indicates a *P* value of <0.05 (one-way ANOVA).

The PfK13 G533S mutation has gained a significant increase in prevalence in the western GMS (16, 19). To determine whether this mutation confers ART partial resistance, we used the CRISPR/Cas9-based marker-free method to introduce the G533S mutation into four parasite lines of distinct origins: 3D7 (Africa), Dd2 (Southeast Asia), GB4 (Ghana), and F09N25, a recently culture-adapted parasite from the western GMS (24). Two out of the three guide RNAs (gRNAs) tested successfully targeted the PfK13 locus, resulting in the desired mutation (Fig. S1; Table S1). After cloning the edited parasite lines, RSA was performed with two clones, each from Dd2^G533S^ and 3D7^G533S^, and one clone each from F09N25^G533S^ and GB4^G533S^. Whereas all the parental lines with a WT PfK13 had similar RSA values (~0.4%) well below the cutoff of <1% for ART partial resistance, all parasite lines engineered with the G533S mutation showed significantly increased RSA rates (Fig. 1; Table S2). Specifically, the Dd2^G533S^ and 3D7^G533S^ lines had ~15% RSA values, with relatively small variations across the clones and replicates (Fig. 1). In comparison, the F09N25^G533S^ and GB4^G533S^ lines presented higher RSA values (23.3% and 20.7%, respectively), albeit with much higher variations among the biological replicates (Fig. 1; Table S2). The G533S mutation showed significantly higher survival rates in all the parasites tested (*P* < 0.05).

Next, we engineered the G533A allele into Dd2 and 3D7 using a similar CRISPR/Cas9 gene editing strategy (Fig. S2; Table S1) since this allele has been reported in three countries (India, Myanmar, and Uganda). The RSA rates, determined for two clones of each parasite line Dd2^G533A^ and 3D7^G533A^, were 1.5% and 1.2%, respectively. These values, although slightly above the 1% cutoff for ART partial resistance, were not significantly different from those of the respective WT parasite strains (Fig. 1).

### PfK13 G533S alters the growth phenotype depending on genetic backgrounds

**Fig 2:**
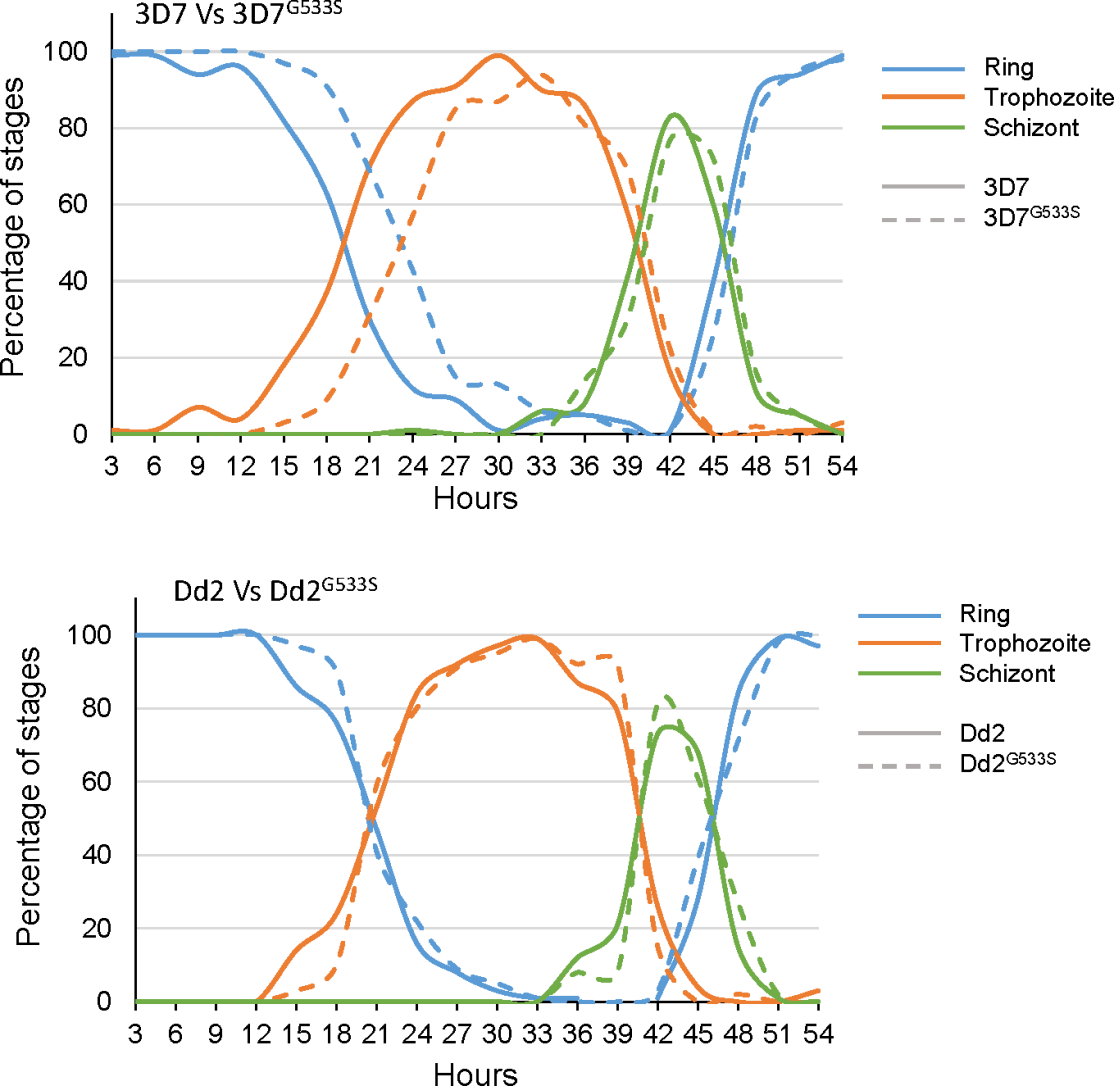
Cell cycle progression for the 3D7^G533S^ and Dd2 ^G533S^ parasites compared to their parental strains. Tightly synchronized parasites with a 3 h window were examined every 3 h, and the percentage of rings, trophozoites, and schizonts was counted. Different colors indicate different asexual stages, while the parental strains and mutants are indicated as solid and dashed lines, respectively.

To study the potential effect of the G533S mutation on parasite growth, we monitored the life cycle progression of tightly synchronized cultures every 3 h. We determined the proportion of different asexual stages at each time point and plotted them to view the difference between Dd2^G533S^ or 3D7^G533S^ and their respective WT parasites. Compared to the WT parasite, the 3D7^G533S^ line showed a prolonged ring stage for approximately 4 h and an accelerated trophozoite stage, eventually finishing the intraerythrocytic developmental cycle at the same time as 3D7 (Fig. 2). However, this prominent extension of the ring stage was not evident in the Dd2 background, with the Dd2^G533S^ and WT Dd2 displaying similar asexual cycle progression curves (Fig. 2).

### PfK13 G533S imparts minimal fitness cost

**Fig 3:**
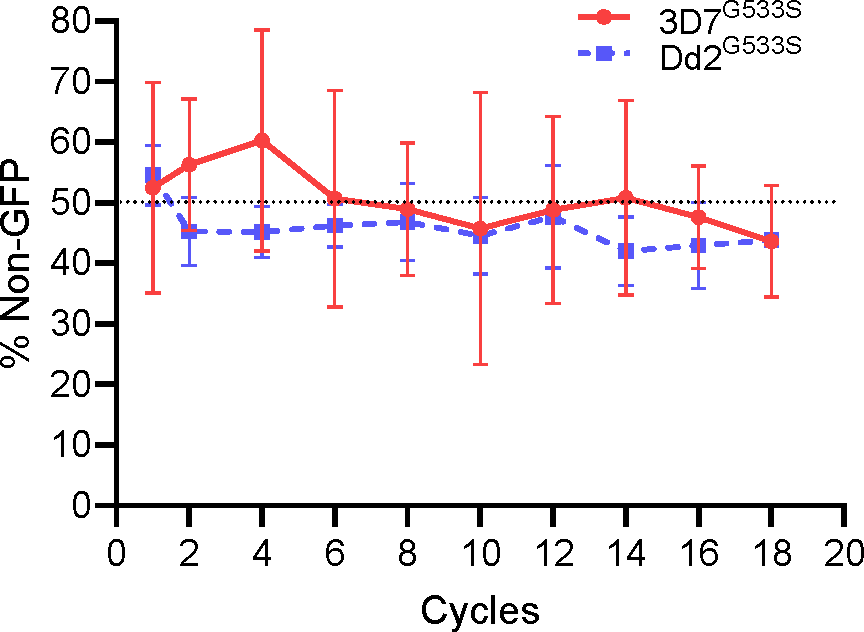
*In vitro* growth competition of 3D7^G533S^ and Dd2 ^G533S^ parasites with their respective parental strains. The 3D7^G533S^ and Dd2 ^G533S^ parasites were mixed in equal proportions with their respective parental lines engineered to express the mNeon GFP reporter, and parasite growth was monitored for 36 days without drug. The proportions of GFP^+^ parasites were measured every 2 days using flow cytometry. Total parasites were determined using the Deep Red MitoTracker staining. The percentage of non-GFP mutant parasites was plotted against the total number of parasites, with the dashed line marking 50%. Each experiment was performed in four biological replicates.

To determine whether the G533S mutation affects the parasite’s fitness in culture, we performed head-to-head growth competition of the G533S mutant and WT parasites. To assist with the assay, we first generated a mNeon GFP reporter line in each of the Dd2 and 3D7 backgrounds (25). For *in vitro* growth competition, we mixed the mutant parasites Dd2^G533S^ and 3D7^G533S^ with their respective mNeon reporter lines in equal proportions and monitored their dynamics for 36 days. As controls, the Dd2^GFP^ and 3D7 ^GFP^ were also mixed with the parental Dd2 and 3D7, respectively. We measured the fractions of non-GFP and GFP parasites using flow cytometry and determined the total parasitemia using the MitoTracker Red staining. Consistent with the mNeon GFP having a minimal effect on parasite fitness (25), the growth of the mNeon GFP parasite lines and their respective WT parasites had negligible differences (Fig. S3). Moreover, we did not observe a significant growth disadvantage of mutant parasites compared to the WT controls. Specifically, both the Dd2^G533S^ and 3D7^G533S^ lines were only marginally reduced to 43% of the total parasite population after 36 days of co-culturing (Fig. 3).

## DISCUSSION

*PfK13* mutations exhibit significant geographic heterogeneity, shaped by the genetic background of local parasite populations and the regional history of antimalarial drug use. These mutations evolve dynamically in response to malaria treatment strategies. For example, the C580Y mutation, which originated in western Cambodia, later spread to Thailand, Vietnam, and southern Laos (26, 27) and became fixed in Cambodia due to the clonal selection of the DHA- and piperaquine-resistant Pailin lineage (7, 28, 29). Similarly, R539T and Y493H mutations are prevalent in Cambodia, while E252Q is more specific to the western Thai border and Myanmar (7, 16, 30). F446I dominates in Myanmar and the China-Myanmar border area (31). Resistance-associated mutations have also been identified in eastern Africa and the Horn of Africa, with R561H, A675V, and C469Y circulating in Rwanda, Uganda, and Tanzania (11, 13, 32), and R622I in Eritrea, Ethiopia, and Sudan (9, 33, 34). The G533S mutation and its alternative alleles were sporadically reported in Asia (Cambodia, India, and Myanmar) (4, 14, 18, 20) and Africa (Zambia, Uganda, and Senegal) (7, 22, 23) until 2014 when its prevalence increased significantly in the China-Myanmar border area (16, 17) and western Thailand bordering Myanmar (19).

Factors specific to these regions, including local malaria dynamics and treatment strategies, may have driven the selection of the G533S mutation. The dissemination of *PfK13* mutations is governed by a balance between the level of resistance conferred and the fitness cost imposed by the mutation. Using gene editing, we demonstrate that G533S can confer partial ART resistance across diverse genetic backgrounds. Growth competition assays showed that G533S imposes minimal fitness cost in both 3D7 and Dd2 parasite strains. This combination of high resistance and low fitness cost likely explains the recent surge in G533S prevalence in the eastern Myanmar border areas compared to its alternative alleles. DHA-PPQ has been adopted as first-line therapy for falciparum malaria in the western GMS. Recent reports from the China-Myanmar border area showed that G533S, along with N458Y and P574L, is associated with delayed parasite clearance and treatment failures in DHA-PPQ efficacy studies conducted over the past decade (17, 19). Since N458Y and P574L impose higher fitness costs, the extensive use of DHA-PPQ may have selected parasites carrying G533S (14, 35).

Since the ring stage of *P. falciparum* is metabolically less active and better equipped to withstand oxidative stress, an extended ring stage helps the parasite to survive under ART pressure (36, 37). Our data suggest that the G533S mutation in the 3D7 background may utilize a similar mechanism to combat ART pressure, though this extended phenotype is absent in Dd2^G533S^, emphasizing the importance of genetic background in the emergence and functional effects of *PfK13* mutations. Since the discovery of the K13 mutations in mediating ART partial resistance, the C580Y mutation has been shown to confer different levels of resistance in different genetic backgrounds (38). Recently, the resistance level mediated by R561H has also been confirmed to be background-dependent (14).

In conclusion, our study confirmed another PfK13 mutation to impart partial ART resistance across multiple genetic backgrounds with a minimal fitness cost. This strong association of G533S with ART partial resistance supports its inclusion as a validated molecular marker for the surveillance of ART resistance.

## MATERIALS AND METHODS

### Parasite culture

Asexual blood-stage parasites were maintained in O^+^ human red blood cells (RBCs) and a humidified 5% CO_2_ incubator at 37°C as previously described (39). Briefly, parasites were grown in RPMI 1640 with 25  mM NaHCO_3_, 11  mM glucose, 25  mM HEPES (pH 7.4), 0.367  mM hypoxanthine, and 5  µg/L gentamicin supplemented with 0.5% AlbuMAX II lipid-rich bovine serum albumin (Thermo Fisher Scientific, MA). Ring-stage parasites were synchronized by 5% d-sorbitol treatment (40).

### Generation of CRISPR-Cas9 edited parasites

CRISPR-Cas9 editing of PfK13 was performed using the pDC2-cam-coCas9-U6-gRNA-hDHFR all-in-one plasmid that contains a *P. falciparum* codon-optimized Cas9 sequence under the *calmodulin* promoter, a human dihydrofolate reductase expression cassette (conferring resistance to WR99210), and a U6 cassette for gRNA expression (41). Three K13 propeller domain-specific gRNAs close to the G533S mutation site were selected (Table S1); each was inserted at the *Bbs*I site. A 492 bp K13 donor template, including the mutation site and the three gRNA sequences, was synthesized by Genewiz (Azenta Life Sciences) and cloned into the pUC-GW-Amp plasmid (Table S3). This K13 donor template included the desired mutation (G533S or G533A), silent binding-site mutations at the Cas9 cleavage site, and a silent shield mutation wherever possible. The K13 donor sequences were then sub-cloned by In-Fusion Cloning (Takara) at the *Eco*RI and *Aat*II sites of the pDC2-cam-coCas9-U6-gRNA-hDHFR plasmid. The final plasmids were sequenced using primers p282, p283, and p35 (Table S1) (14). A schematic showing the method of plasmid construction is shown in Fig. S1.

### Generation of K13 G533S gene-edited lines

Gene-edited lines were generated by electroporating fresh RBCs with 50–100 μg of plasmid DNA resuspended in Cytomix, followed by incubation with late-stage parasites at 0.2% final parasitemia (42). Twenty-four hours later, transfected parasites were selected with WR99210 (Jacobus Pharmaceuticals) for 7–10 days. Parasite cultures were monitored by microscopy for parasite appearance for 6–8 weeks. To verify successful gene editing, the K13 locus was amplified using K13Fseqg533s/K13Rseqg533s (Table S1), and the PCR products were sequenced by Sanger sequencing. Successfully edited parasites were cloned by limiting dilution to obtain single parasite clones (Fig. S1).

### Ring-stage survival assays

RSAs were performed as previously described (3, 35). Briefly, a 75% Percoll (Sigma-Aldrich) gradient was used to purify late-stage schizonts, which were allowed to rupture and invade fresh RBCs for 3 h. Three hours later, 5% sorbitol was used to eliminate the remaining schizonts. These 0–3 h ring-stage parasites at 1% parasitemia and 2% hematocrit were exposed to 700  nM DHA or 0.1% dimethyl sulfoxide (DMSO) for 6 h. The cultures were then washed with fresh media, and viable parasites were evaluated 66 h later by counting ∼10,000 RBCs. Ring-stage survival rates were expressed as the ratios of viable parasites in DHA vs DMSO samples, with 1% considered the threshold for ART partial resistance (3).

### Phenotype analysis

To monitor cell cycle progression, late schizont-stage parasites were purified from tightly synchronized cultures using 75% Percoll and allowed to invade fresh RBCs for 3 h as described above. Cultures were incubated in 24-well plates at 1% parasitemia and 2% hematocrit. The proportion of rings, trophozoites, and schizonts was recorded every 3 h using Giemsa-stained thin smears until 60 h (43). Rings were recorded as small ring-like parasites with blue cytoplasm, while larger parasites with dark pigment (hemozoin) were considered trophozoites. Parasites with more than two nuclei were counted as schizonts.

### *In vitro* growth competition assay

We generated mNeon GFP reporter lines in Dd2 and 3D7 using the plasmid pDC2-coCas9-*pare*-BSD-nNeonGreen and blasticidin S deaminase (BSD) selectable marker as previously reported (25). To determine the potential fitness cost associated with G533S, we performed a mixed-culture competition assay by mixing the Dd2 ^G533S^ or 3D7^G533S^ mutant line with Dd2^GFP^ or 3D7^GFP^, respectively, in a 1:1 ratio at a 3% ring-stage parasitemia. Dd2^GFP^ or 3D7^GFP^ was also mixed with Dd2 or 3D7 in equal proportion as control. Cultures were maintained for 36 days. One-fourth of the parasites were used for flow cytometry analysis every 2  days. Cultures were diluted to half and replenished with fresh blood twice a week. The ratio of GFP-positive (WT control) and the total number of parasites measured by 100 nM MitoTracker Deep Red staining was recorded using flow cytometry for 36  days. Four independent experiments were performed in duplicate, and the proportion of GFP-negative (mutant) parasites was plotted over time. Each time, a total of 100,000 events were read per well.

### Statistical analysis

Statistical analysis was performed using the GraphPad Prism (v5) program. A non-parametric Wilcoxon matched-pairs test or one-way ANOVA was used to compare the mean values between treatment groups. Differences were considered significant at a *P* value of <0.05.

## SUPPLEMENTAL MATERIAL

Supplemental material aac.00162-25-s0001.pdf Fig. S1 to S3; Tables S1 to S3.

ASM does not own the copyrights to Supplemental Material that may be linked to, or accessed through, an article. The authors have granted ASM a non-exclusive, world-wide license to publish the Supplemental Material files. Please contact the corresponding author directly for reuse.
