# Supplementary material for: The PfK13 G533S mutation confers artemisinin partial resistance in multiple genetic backgrounds of Plasmodium falciparum
Source: Antimicrob Agents Chemother. 2025 May 27;69(7):e00162-25. doi: 10.1128/aac.00162-25 (PMC12217484; doi:10.1128/aac.00162-25)
Supplement: Supplemental material — Fig. S1 to S3; Tables S1 to S3. [file aac.00162-25-s0001.pdf]

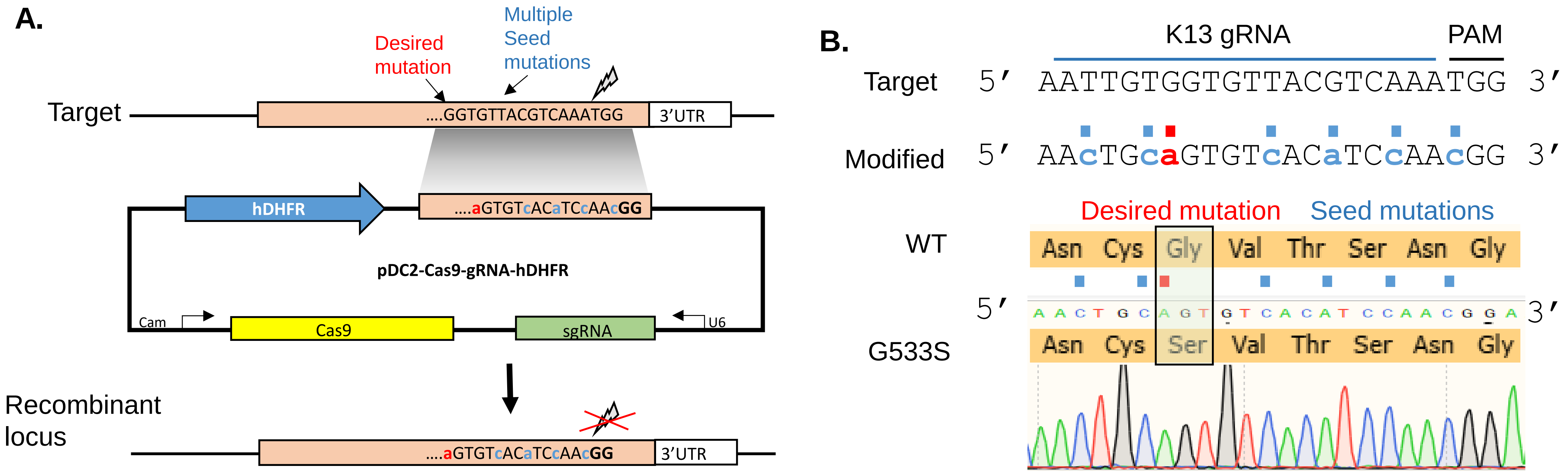

Figure S1. Schematic illustrating the marker-free CRISPR/Cas9 editing of the K13 locus to introduce the G533S mutation. (A) Donor fragment of 0.5 kb was designed with the desired point mutation in the center (shown in red) and additional silent mutations (shown in blue) in the gRNA target sites marked by a lightning bolt. These additional mutations are important to prevent cleavage of the modified locus as well as the plasmid. The donor and the gRNA are cloned into the all-in-one plasmid pDC2-cam-Cas9-U6-hDHFR that expresses a Cas9 cassette driven by the cam promoter, a gRNA under U6 promoter, and a selectable marker, hDHFR. **(B)** Target locus recognized by sgRNA^{K13}:Cas9 including the 20-nucleotide guide sequence and the PAM. Modified sequence shows the desired mutation (red) that results in G533S modification. Since a silent mutation in PAM was not possible, multiple shield mutations (blue) were introduced in the gRNA. Chromatogram is shown at the bottom with WT and mutated sequences (G533S). The desired nucleotide and protein mutations are highlighted. Primers used for cloning and sequencing are described in Table S1.

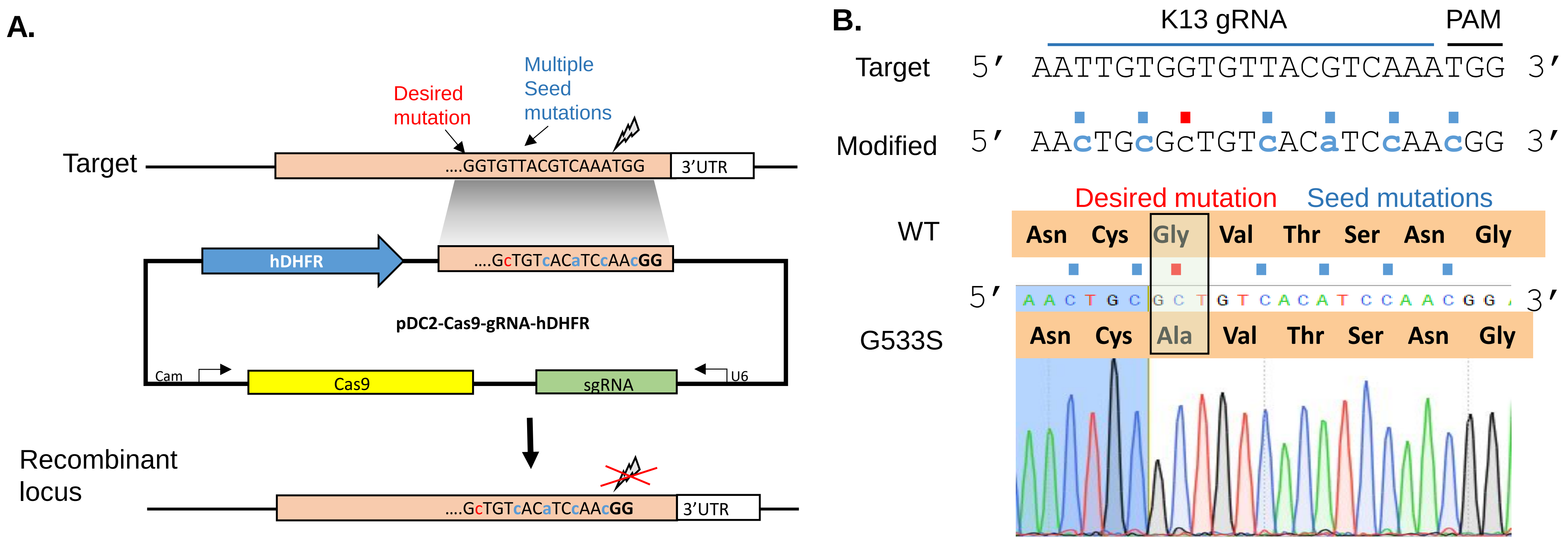

Figure S2. Schematic illustrating the marker-free CRISPR/Cas9 editing of the K13 locus to introduce the G533A mutation. (A) Donor fragment of 0.5 kb was designed with the desired point mutation in the center (shown in red) and additional silent mutations (shown in blue) in the gRNA target sites marked by a lightning bolt. These additional mutations are important to prevent cleavage of the modified locus as well as the plasmid. The donor and the gRNA are cloned into the all-in-one plasmid pDC2-cam-Cas9-U6-hDHFR that expresses a Cas9 cassette driven by the cam promoter, a gRNA under U6 promoter, and a selectable marker, hDHFR. **(B)** Target locus recognized by sgRNA^{K13}:Cas9 including the 20-nucleotide guide sequence and the PAM. Modified sequence shows the desired mutation (red) that results in G533A modification. Since a silent mutation in PAM was not possible, multiple shield mutations (blue) were introduced in the gRNA. Chromatogram is shown at the bottom with WT and mutated sequences (G533A). The desired nucleotide and protein mutations are highlighted. Primers used for cloning and sequencing are described in Table S1.

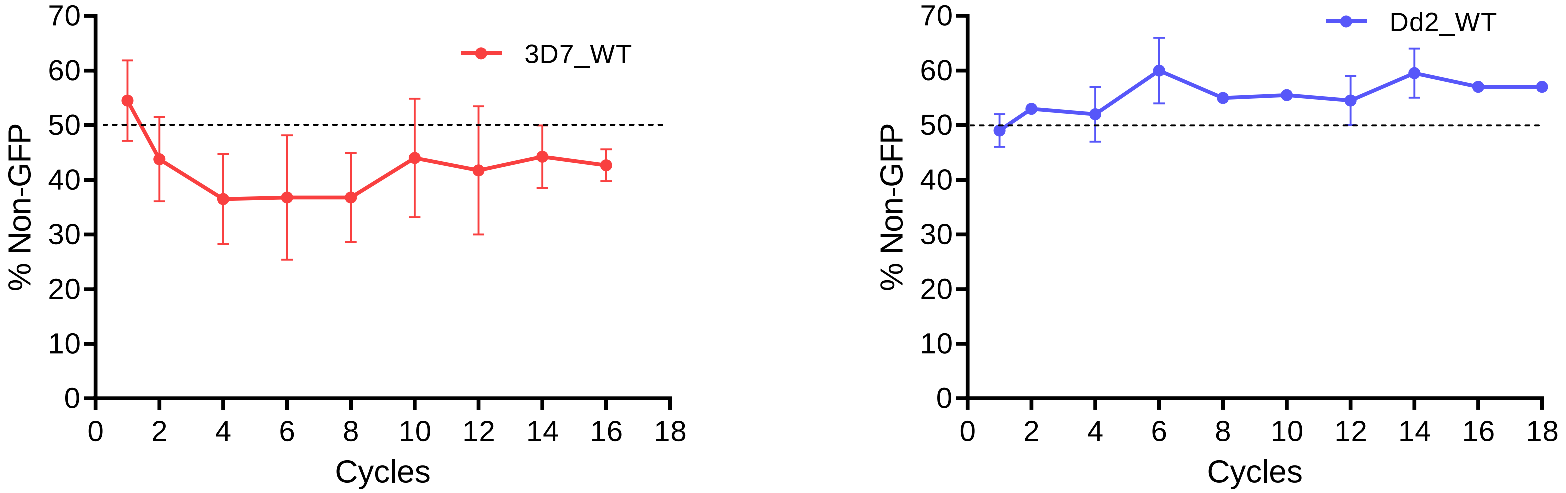

Figure S3. *In vitro* growth competition assays for GFP control parasites 3D7-GFP and Dd2-GFP with their respective wild-type (WT) parasites. The assay was started by combining 3D7-GFP (or Dd2-GFP) in equal proportion with the WT 3D7 (or Dd2) parasites. Parasites with GFP fluorescence and total parasites (Deep Red MitoTracker) were measured every 2 days using flow cytometry. Each experiment was performed in at least 2 biological replicates over a period of a month.

Table S1. Primers used for K13 editing and sequencing.

Primer name	Primer sequence
K13_G533s_ML2_1Oligo1	TATTATACCTAGAAGAAATAATTG
K13_G533s_ML2_1Oligo2	AAACCAATTATTTCTTCTAGGTAT
K13_G533s_ML2_2Oligo1	TATTAATTGTGGTGTTACGTCAAA
K13_G533s_ML2_2Oligo2	AAACTTTGACGTAACACCACAATT
K13_G533s_ML2_3Oligo1	TATTACACCACAATTATTTCTTCT
K13_G533s_ML2_3Oligo2	AAACAGAAGAAATAATTGTGGTGT
P35	aagcaccgactcggtgccac
p282	AACATATGTTAAATATTTATTTCTC
p283	AGGGTTATTGTCTCATGAGCGG
K13Fseqg533s	GTGAGTTATTTAGAATTATACTTA
K13Rseqg533s	GGTACACCATTTAGAAATTGC

Table S2. Survival rates of parasite clones.

	R1	R2	R3	R4
Dd2_G533S C-1	17.72	21.48	16.23	11.68
Dd2_G533S C-2	14.04	25.86	13.26	13.63
Dd2_G533A C-1	3.08	0.80	1.90	
Dd2_G533A C-2	0.80	1.35	1.07	
Dd2	0.00	0.10	0.35	
3D7_G533S C-1	24.13	17.99	7.24	
3D7_G533S C-2	15.75	6.84	16.33	
3D7_G533A C-1	2.39	0.00	1.20	
3D7_G533A C-2	2.50	0.00	1.27	
3D7	0.61	0.20	0.10	
F09N25_G533S	32.87	13.63		
F09N25	0.00	0.00	0.30	
GB4_G533S	14.48	41.81	5.72	
GB4	0.00	0.00	0.20	

The RSA value of each clone was determined in 3-4 biological replicates.

Table S3. Donor template fragments with G533S and G533A mutations for CRISPR/Cas9 editing of *PfK13*.

PfK13 WT sequence	TAGAATATTTAAATTCGATGGAATTATTAGATATTAGTCAACAATGCTGGCGTATGTGTACACCTATGTCTACCAAAAAA<br>GCTTATTTTGGAAGTGCTGTATTGAATAATTTCTTATACGTTTTTGGTGGTAATAACTATGATTATAAGGCTTTATTTGA<br>AACTGAGGTGTATGATCGTTTAAGAGATGTATGGTATGTTTCAAGTAATTTAAATATACCTAGAAGAAATAATTGTGGTG<br>TTACGTCAAATGGTAGAATTTATTGTATTGGGGGATATGATGGCTCTTCTATTATACCGAATGTAGAAGCATATGATCAT<br>CGTATGAAAGCATGGGTAGAGGTGGCACCTTTGAATACCCCTAGATCATCAGCTATGTGTGTTGCTTTTGATAATAAAAT<br>TTATGTCATTGGTGGAACTAATGGTGAGAGATTAAATTCTATTGAAGTATATGAAGAAAAAATGAATAAATGGGAACAAT
**G533S** (GGT to aGT)	TAGAATATTTAAATTCGATGGAATTATTAGATATTAGTCAACAATGCTGGCGTATGTGTACACCTATGTCTACCAAAAAA<br>GCTTATTTTGGAAGTGCTGTATTGAATAATTTCTTATACGTTTTTGGTGGTAATAACTATGATTATAAGGCTTTATTTGA<br>AACTGAGGTGTATGATCGTTTAAGAGATGTATGGTATGTTTCAAGTAATTTAAATATcCCcAGgAGgAAcAAcTGcaGTG<br>TcACaTCcAAcGGaAGAATTTATTGTATTGGGGGATATGATGGCTCTTCTATTATACCGAATGTAGAAGCATATGATCAT<br>CGTATGAAAGCATGGGTAGAGGTGGCACCTTTGAATACCCCTAGATCATCAGCTATGTGTGTTGCTTTTGATAATAAAAT<br>TTATGTCATTGGTGGAACTAATGGTGAGAGATTAAATTCTATTGAAGTATATGAAGAAAAAATGAATAAATGGGAACAAT
**G533A** (GGT to GcT)	TAGAATATTTAAATTCGATGGAATTATTAGATATTAGTCAACAATGCTGGCGTATGTGTACACCTATGTCTACCAAAAAA<br>GCTTATTTTGGAAGTGCTGTATTGAATAATTTCTTATACGTTTTTGGTGGTAATAACTATGATTATAAGGCTTTATTTGA<br>AACTGAGGTGTATGATCGTTTAAGAGATGTATGGTATGTTTCAAGTAATTTAAATATcCCcAGgAGgAAcAAcTGcGcTG<br>TcACaTCcAAcGGaAGAATTTATTGTATTGGGGGATATGATGGCTCTTCTATTATACCGAATGTAGAAGCATATGATCAT<br>CGTATGAAAGCATGGGTAGAGGTGGCACCTTTGAATACCCCTAGATCATCAGCTATGTGTGTTGCTTTTGATAATAAAAT<br>TTATGTCATTGGTGGAACTAATGGTGAGAGATTAAATTCTATTGAAGTATATGAAGAAAAAATGAATAAATGGGAACAAT

The wild-type sequence is included here for comparison. The location of the gRNA1 (ATACCTAGAAGAAATAATTG) is highlighted in green. Mutations are highlighted in red, and lowercase letters indicate multiple shield mutations. The other two guide RNAs are:
gRNA2 (AATTGTGGTGTTACGTCAAA) and gRNA3 (ACACCACAATTATTTCTTCT).
